# Assessing the spread of COVID-19 in Brazil: Mobility, morbidity and social vulnerability

**DOI:** 10.1371/journal.pone.0238214

**Published:** 2020-09-18

**Authors:** Flávio C. Coelho, Raquel M. Lana, Oswaldo G. Cruz, Daniel A. M. Villela, Leonardo S. Bastos, Ana Pastore y Piontti, Jessica T. Davis, Alessandro Vespignani, Claudia T. Codeço, Marcelo F. C. Gomes

**Affiliations:** 1 Escola de Matemática Aplicada, Fundação Getulio Vargas, Rio de Janeiro, Brazil; 2 Núcleo de Métodos Analíticos para Vigilância em Saúde Pública, Rio de Janeiro, Brazil; 3 Programa de Computação Científica, Fundação Oswaldo Cruz, Rio de Janeiro, Brazil; 4 London School of Hygiene and Tropical Medicine, London, United Kingdom; 5 Laboratory for the Modeling of Biological and Socio-technical Systems, Northeastern University, Boston, MA, United States of America; 6 ISI Foundation, Turin, Italy; Columbia University, UNITED STATES

## Abstract

Brazil detected community transmission of COVID-19 on March 13, 2020. In this study we identified which areas in the country were the most vulnerable for COVID-19, both in terms of the risk of arrival of cases, the risk of sustained transmission and their social vulnerability. Probabilistic models were used to calculate the probability of COVID-19 spread from São Paulo and Rio de Janeiro, the initial hotspots, using mobility data from the pre-epidemic period, while multivariate cluster analysis of socio-economic indices was done to identify areas with similar social vulnerability. The results consist of a series of maps of effective distance, outbreak probability, hospital capacity and social vulnerability. They show areas in the North and Northeast with high risk of COVID-19 outbreak that are also highly socially vulnerable. Later, these areas would be found the most severely affected. The maps produced were sent to health authorities to aid in their efforts to prioritize actions such as resource allocation to mitigate the effects of the pandemic. In the discussion, we address how predictions compared to the observed dynamics of the disease.

## Introduction

As of 21 March 2020, the pandemic of COVID-19 had reached 184 countries with 266,073 confirmed cases and 11,184 deaths, globally [1]. The first imported case of COVID-19 was confirmed in Brazil on February 26, 2020, in the city of São Paulo [2], only two months after the alert on COVID-19 went off in China. At this date, all twenty seven federative units had reported suspect cases of COVID-19 infection, while 16 states and the Federal District had confirmed 1128 cases (11,278 under investigation) and 18 deaths [3].

São Paulo and Rio de Janeiro states identified virus community transmission on March 13, 2020 [4–6]. Just four days later, 240 cases were confirmed in São Paulo, with 4 deaths [3]. In Rio de Janeiro, 45 cases had been confirmed with no reported death. São Paulo and Rio de Janeiro states hold the most populous metropolitan areas of Brazil, where a large fraction of the population live in crowded neighborhoods with poor housing and low income. They are the country’s main hubs for national and international transportation. It is known that other pathogens such as Influenza A H1N1, in 2009, have been introduced in the country through these hubs [7].

In March 2020, when the epidemic was still confined to Rio de Janeiro and São Paulo, we estimated the pattern of COVID-19 spreading risk within Brazil, taking the states of Rio de Janeiro and São Paulo as the starting points. In our analysis, we considered a worst case scenario in which there would be no implementation of mobility restrictions. At that moment, mobility restrictions and social distance were starting to be adopted, but it was not clear whether the population would adhere to them.

Brazil is a continental country with strong spatial heterogeneities in terms of demography, age distribution, access to public health, and socioeconomic indexes. Because of these inequalities, the COVID-19 epidemic should impact these populations differently. To identify regions with high geographical and social vulnerability, we proposed a classification scheme based on three main criteria: population mobility, socio-demographic-economic characteristics, and the available health care infrastructure in terms of hospital capacity.

## Materials and methods

### Data

Brazil is divided into 558 micro-regional administrative units, with population sizes varying from 13 million people in the metropolitan area of São Paulo to 2, 703 in Fernando de Noronha island, in Pernambuco.

To measure mobility intensity between micro-regions, we used daily air travel statistics from the Official Airline Guide (OAG) [8]. This dataset contains the number of travels per origin-destination airport. Data on shorter distance pendular travels motivated by work and study activities were obtained from the 2010 national census (IBGE) [9].

Demographic data from the 2000 and 2010 national censuses [9] were used to project the population per age group in 2020 using a geometric growth model. Socioeconomic indicators were obtained from the same source: infant mortality, life expectancy, GINI index, human development index (education, longevity and income), proportion of individuals below the poverty threshold, proportion below the extreme poverty threshold, 25% percentile income, percentage of urban population, percentage of the population in households with piped water, % of population with insufficient water supply and precarious sewage disposal, and percentage of individuals in households without electricity.

Data on health care capacity per micro-region were obtained from DataSUS [10]. From these data, we calculated the number of standard hospital beds and number of complementary beds (Intensive Care Unit and Intermediate Unit) available for each micro-region [10], aggregating those from the public sector (SUS) and private (non-SUS) sector. The final indicator is given by 10, 000 inhabitants.

Number of COVID-19 cases notified per day was obtained from the site brasil.io/dataset/covid19/casofull. This site aggregates official notification data reported by each state.

### Effective distance

To assess the probability of COVID-19 spreading within Brazil, in the absence of mobility restrictions, we first calculated the effective distance (*E*_*f*_(*i*,*j*)) between micro-regions using the air travel data. Due to the continental size of Brazil, daily interstate mobility between major urban centers is mainly composed by air travel. Therefore, interstate dispersion of the disease during the initial phase of the epidemic is assumed to be driven mainly by air-travel. We computed the effective distance, *E*_*f*_(*i*,*j*), between each micro-region and the two COVID-19’s hotspots, Rio de Janeiro and Sao Paulo. *E*_*f*_(*i*,*j*) is a measure of proximity between micro-regions *i* and *j* created by the flow of travellers. This metric is known to be strongly correlated with the time it takes to import infectious diseases into new territories from a well-defined origin, particularly for diseases with direct transmission [11, 12]. We computed *E*_*f*_ from São Paulo and Rio de Janeiro separately in order to assess the potential contribution of each one.

To facilitate comparison between different scenarios, we also calculated a relative effective distance (*e*_*f*_), by dividing *E*_*f*_ by the distance to the nearest destination: *e*_*f*_(*i*,*j*) = *E*_*f*_(*i*,*j*)/min_*j*_{*E*_*f*_(*i*,*j*)}. This information was mapped into micro-region origin-destination pairs by summing over the corresponding airports serving each micro-region based on its municipality of reference.

### Outbreak probability

To calculate the probability of outbreak in each micro-region *m*, we used the standard expression: $p_{epi}=1-{\left(1/R_{0}\right)}^{I_{m}}$ [13]. This expression comes from the stochastic SIR model, where the probability of extinction of an outbreak is given by ${\left(1/R_{0}\right)}^{I_{0}}$ where *I*_0_ is the initial number of infected when *R*_0_ > 1. Thus, the complement of this probability is the probability of the epidemic taking hold in the population (see [13], p.106 for a derivation). The prevalence of infection, *I*_*m*_, is estimated as *I*_*m*_ = *kτ* ∑_*i*_ *f*_*i*,*m*_ *I*_*i*_/*N*_*i*_, where *f*_*i*,*m*_ is the number of travelers with COVID-19 arriving from micro-region *i* to micro-region *m* while *I*_*m*_ is the product of the fractional prevalence *I*_*i*_/*N*_*i*_ times the infection duration *τ* (assumed equal for all infected) and a scaling parameter *k*, to account for the number of undetected asymptomatic individuals participating in the transmission. The parameter *R*_0_ is the basic reproduction number [13]. For the purpose of the results shown, we set *R*_0_ = 2.5, which is compatible with previous studies [14–17]. S1 Fig shows how variations in the parameters of the equation affect the probability of outbreak. For *R*0 > 2, probability of outbreak is likely given the arrival of at least one infected individual.

We computed the outbreak probability per micro-region using two consecutive time windows representing two generations of spread:

#### First generation of outbreaks

We assumed that community transmission has been reached when the count of cases was 100 cases. We selected only Rio de Janeiro and São Paulo as the source municipalities which were the two initial COVID-19 hotspots. Prevalence of infection was calculated by multiplying the notification count by an expansion factor *k* = 10 to take into account asymptomatic and unreported cases [18]. This number is then multiplied by the average duration of infection *τ* = 8 days [19], resulting in 8000 prevalent infections (infected × infection duration) in each of the two cities of origin.

The number of travelers per day between micro-regions was computed by adding the number of air travellers (used to calculate effective distance) and the number of pendular commuters Census [9]. The inclusion of pendular mobility was important to allow spreading between geographically close micro-regions, while still preserving data-driven flow estimates.

#### Second generation of outbreaks

In this scenario, we assumed that enough time had passed so that all micro-regions with *p*_*epi*_ ≥ 0.5 in the first scenario actually had developed community transmission (epidemic). In each of the new hotspots, the prevalence is set to the same level adopted before for Rio de Janeiro and São Paulo micro-regions. Then we computed *p*_*epi*_ again, to identify the micro-regions most likely to develop outbreaks in this second round. These generations of outbreaks should not be confused with generations of infections within a city.

The above mentioned scenarios do not take into account any intervention affecting mobility, nor demographic and environmental effects that may play a role on the magnitude of *R*_0_.

#### Social vulnerability

Partitioning cluster analysis (k-medoid) was performed to identify micro-regions with similar social vulnerability. This method is a more robust variation of the standard k-means. The first step was the removal of highly linearly correlated variables (Pearson’s correlation > 0.8): infant mortality, human development index (longevity and income), proportion of individuals below the poverty threshold, 25% percentile income, percentage of urban population. The following ones remained: life expectancy, GINI index, education component of the human development index, proportion of the population living below the extreme poverty threshold, percentage of urban population, percentage of the population in households with piped water, percentage of population with insufficient water supply and precarious sewage disposal, and percentage of individuals without electricity. The elbow method was used to estimate the number of clusters [20, 21]. More information on this analysis can be found in the S2 Supplement Material. The analysis was done using the R environment [22], packages cluster [23], corrplot [24], FactoMineR [25], factoextra [20].

## Results

### Effective distance in the absence of travel restrictions

**Fig 1:**
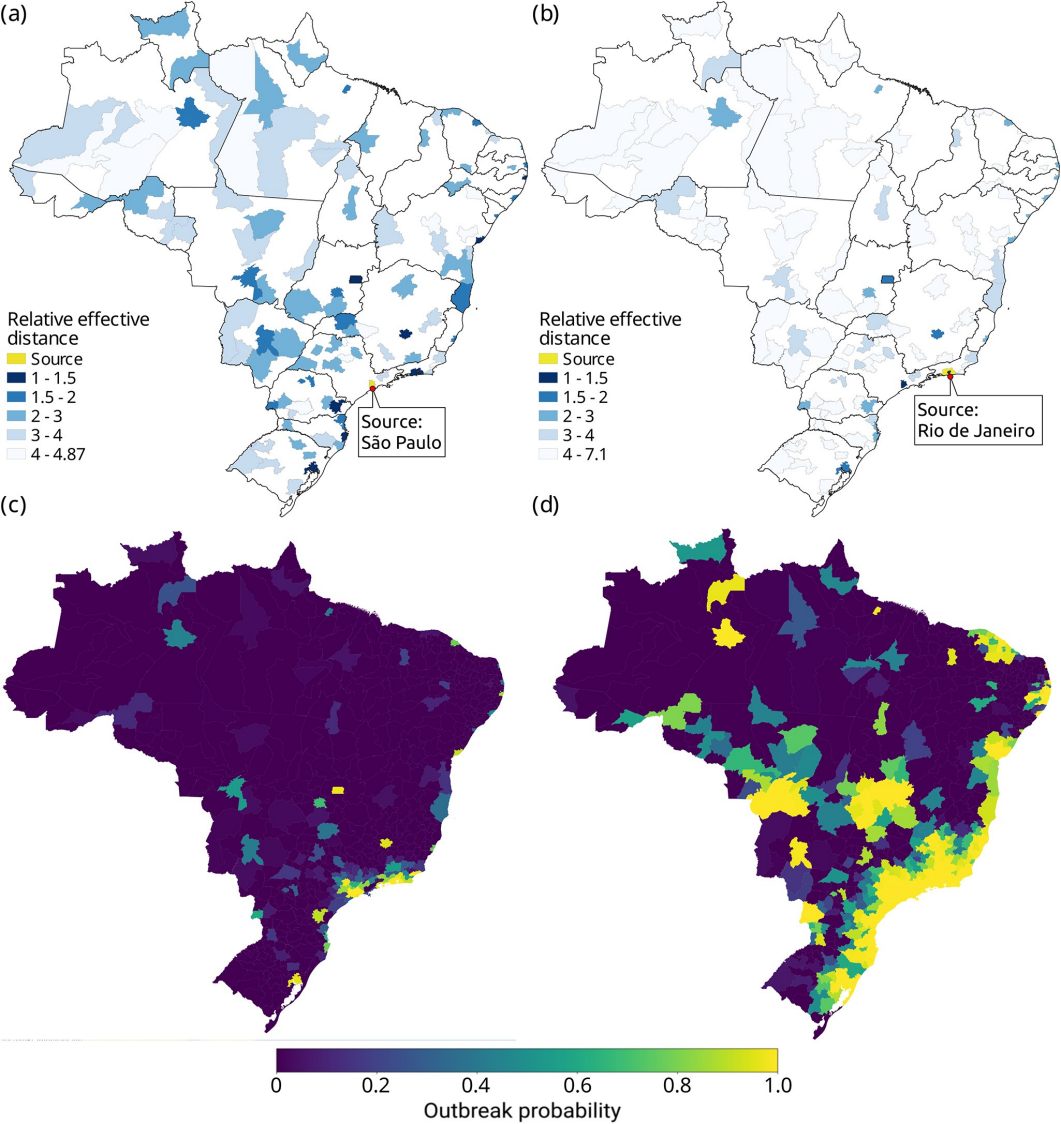
Effective distance from the two initial COVID-19 hotspots in Brazil. (a) Relative effective distance (*e*_*f*_) of Brazilian micro-regions from São Paulo based on airline network in the absence of travel restrictions. (b) The same from Rio de Janeiro, with blue gradient from closest (dark blue) to farthest (light blue) destinations, limited to those present on the airline network. (c) Probability of COVID-19 outbreak per micro-region as Rio de Janeiro and São Paulo sustain high prevalence of infection. (d) Second round of outbreaks after the micro-regions infected in (c) begin to contribute cases, with a gradient from dark purple (*p* = 0) to bright yellow (*p* = 1.0).

Fig 1(a) and 1(b) show the relative effective distance of the Brazilian micro-regions from São Paulo and Rio de Janeiro measured by the typical travel movement by air. The majority of state capitals are among the closest areas, together with some important touristic destinations (such as Foz do Iguaçu/Paraná and Porto Seguro/Bahia). Important urban and industrial centers such as Itajaí/Santa Catarina and Uberlândia/Minas Gerais are also close. São Paulo shows a more central position than Rio de Janeiro, evidenced by the larger proportion of closer destinations, suggesting that an uncontrolled hotspost of COVID-19 in Sao Paulo produces a greater risk for earlier and widespread case exportation to other states.

### Probability of outbreak

Fig 1(c) shows the probability of COVID-19 outbreak per micro-region triggered by the increased prevalence of COVID-19 in Rio de Janeiro and São Paulo, in the absence of travel restrictions. The most likely micro-regions to develop an outbreak are the geographic neighboring regions of São Paulo and Rio de Janeiro, all state capitals of the South and Southeast regions (Belo Horizonte, Vitória, Curitiba, Florianópolis and Porto Alegre), plus Brasilia, Recife and Salvador, among others. A more complete list is found in the S1 Supporting Information.

Fig 1(d) shows the probability of outbreak in a secondary round of propagation, conditioned to the establishment of transmission in the micro-regions with highest risk (*p* > 0.5) during the first phase. In this second moment, the establishment of COVID-19 transmission is very likely along the coast, from Porto Alegre (in the South) to Salvador (Northeast). Other high risk areas are the neighboring areas of Recife and Fortaleza (Northeast), the neighboring areas of Foz do Iguaçu, in the western border of Paraná state, and the neighboring areas of Cuiabá, Brasilia, and Goiânia in the Center-West.

### Social vulnerability

We identified five classes of social vulnerability, tentatively ordered from A (least vulnerable) to D-E (most vulnerable). Table 1 shows the averages of the socioeconomic indexes for each class, from which we propose the following interpretation. The Supporting information file S2 has more detailed description of the analysis.

**Table 1:** Mean value of the descriptors in the five classes of social vulnerability in Brazil.

Class	life expect.	GINI	poverty	water	sewage	electricity	urban	HDI edu.
A	75.0	0.46	2.16	95.9	1.30	0.39	0.79	0.64
B	74.2	0.49	5.99	89.15	4.10	1.59	0.64	0.57
C	70.9	0.52	20.38	80.46	15.266	4.27	0.56	0.50
D	69.96	0.53	25.72	61.88	24.14	4.03	0.49	0.47
E	70.82	0.60	31.55	70.09	41.15	16.84	0.50	0.45

Life expect. = life expectancy (age), GINI, poverty = % living in extreme poverty, water = % individuals without access to piped water, sewage = % population with insufficient water supply and precarious sewage disposal, electricity = % individuals in households without electricity, urban = % living in cities.

*Class A*. Mostly urban micro-regions, with above-average life expectancy, with comparatively less social inequality, less population living in extreme poverty, better access to water supply and sewage disposal services, higher education. They are the largest cities and in the central region.

*Class B*. Very similar to A in life expectancy. Still more urban, but with more population living in extreme poverty (mean = 5%). Inequality indexes and infrastructure are worse in comparison to A, but still above average. These are found in the South, Southeast, and Central regions.

*Class C*. Mixture or urban and rural populations. In comparison to A and B, they have significantly lower life expectancy, significantly high poverty and less infrastructure. They are the most urbanized areas of the Northeast region. Manaus, capital of the Amazonas state in the North region, is also in this category.

*Class D*. Predominance of rural populations, high inequality, low HDIedu, poor access to water and sewage services, but with access to electricity. These are mainly located in the dry *Caatinga biome* area of the Northeast.

*Class E*. Predominantly rural regions in the Amazon. Low HDIedu, poor access to treated water, sewage disposal, and electricity.

**Fig 2:**
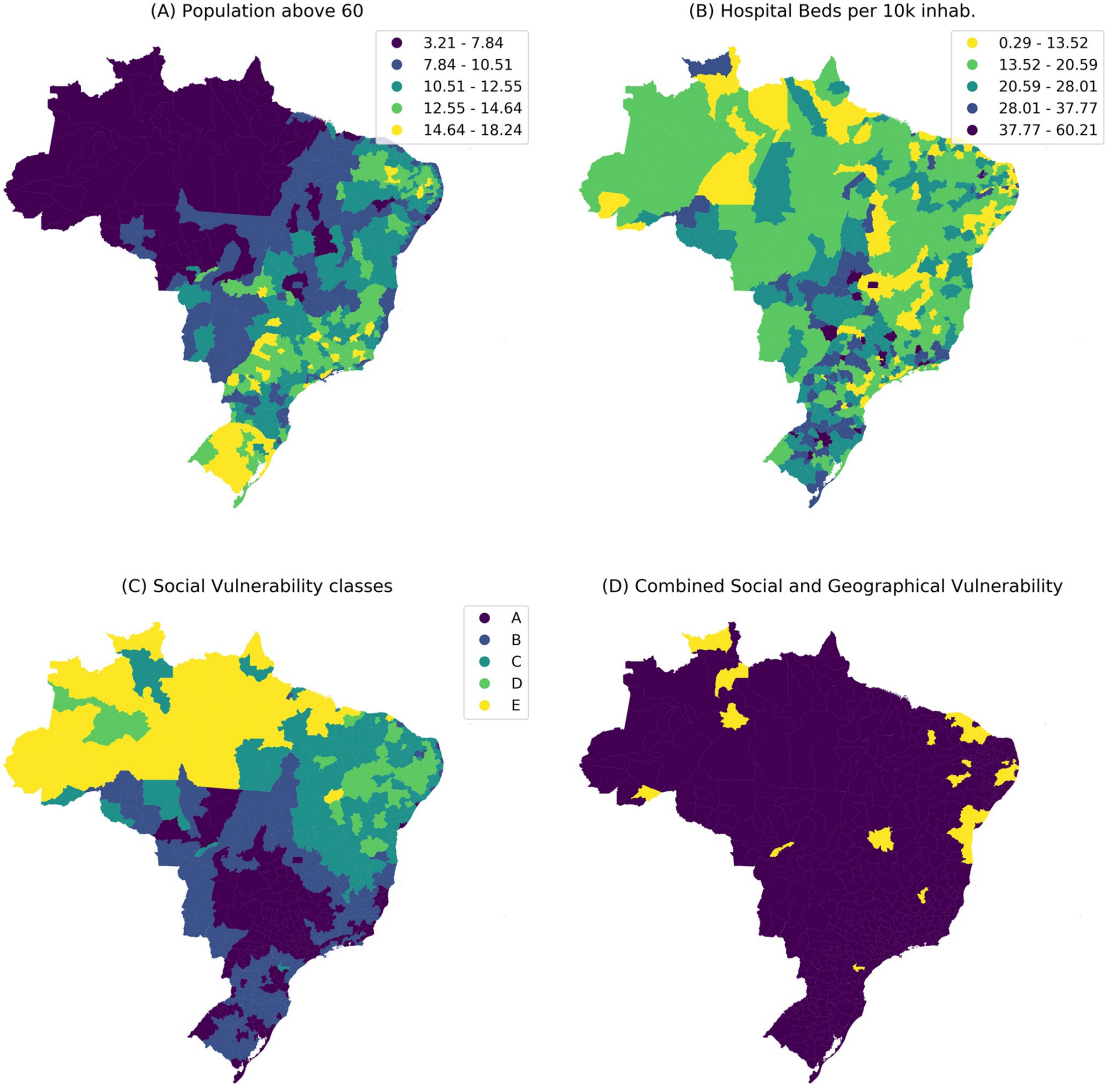
Vulnerability panel. (Top left) Percentage of population above 60 years old (Top right) Hospital capacity as number of hospital beds per 10 per 10,000 individuals; (Bottom left) classification of homogeneous areas in terms of socio-economic vulnerability. D and E are the most vulnerable; (Bottom Right) selection of micro-regions with high probability of imminent COVID-19 epidemics and high social vulnerability.

Fig 2 shows four maps that synthesize the main vulnerabilities to COVID-19 in Brazil in March 21th. Fig 2(A) shows a strong difference in age structure, with the proportion of individuals with 60 or more years of age varying from only 3% in the North to more than 15% in the Southern part (Fig 2(A).

Fig 2(C) shows micro-regions with similar social vulnerability indexes. Micro-regions in the C, D and E classes are the most vulnerable. They are located mostly in the Northeast and North regions. As expected, higher life expectancy is associated with better living conditions, significantly concentrated in the Southern portion of the country. Hospital capacity is very heterogeneous, the best capacity found in large metropolitan areas. There are under-equipped micro-regions throughout the country but they concentrate in the North and Northeast.

Fig 2(D) highlights the micro-regions with high probability of imminent outbreak (*p*_*out*_ > 0.5 on the second wave) and high social vulnerability. They are important targets for immediate attention in terms of local socio-economic factors (S1 File).

## Discussion

In the absence of strong mobility restrictions, the fast establishment of COVID-19 outbreaks in the larger urban areas of the country was found to be highly likely, with subsequent spread to their vicinity municipalities. The time scale of this spread is not explicitly represented in the model, but it was expected to be a matter of a few weeks considering the short serial interval of this infection. The implementation of mobility restrictions could delay this spread but it was unlikely to change the routes of the travelling wave. The analysis of COVID-19 spread using the mobility flows and probabilities of the epidemic taking off, were subjected to uncertainties in both mobility and epidemiological parameters available at the time they were calculated. Nevertheless their utility at the time was not diminished by this uncertainty since the parameters were chosen to represent a somewhat extreme scenario, which the country could still strive to avoid. Other uncertainties expected to limit the accuracy of the results were the lack of an accurate value for the infectious period of COVID-19 and the lack of knowledge about early changes in mobility resulting from news of the pandemic already circulating through the country.

Social distancing was one of the main strategies put in place to delay transmission. This was expected to be difficult to achieve in areas with high social vulnerability where poor living conditions make it difficult to adhere to hygiene and isolation protocols. Even in the *A* vulnerability class, the success of this strategy requires adaptation to cope with the large inequality within the municipalities of each micro-region. The classification proposed here was developed to help to tailor the mitigation protocols to the needs and possibilities of the different regions.

It was clear the great heterogeneity in hospital capacity across the country. The median number of hospital beds was 19 per 10,000, but 5% of the micro-regions have only 6 beds per 10,000. This disparity poses an important challenge for resource allocation, and should be addressed specially in those municipalities combining high probability to early spread, relatively high percentage of individuals above 60 years old, and limited number of hospital beds per 10,000 people. All discussions about the time to overload of the health system should take into consideration the regular level of occupancy of hospital beds in each region, which was not available for this analysis.

The regions in the North and Northeast of the country were those identified with the highest socioeconomic vulnerabilities. These areas were expected to suffer above average burden if measures were not taken quickly, since the eventual disease spread would add to struggles already present in those populations.

All the analyses proposed and applied here focused on being able to rank regions in terms of the seriousness of the health crisis looming over them. As such, their main limitation is that they yield relative results, allowing for optimal resource allocation, but not for accurately predicting number of cases and deaths. Further exploration of this model could include Monte Carlo methods to identify uncertainties regarding different trajectories of disease spread in the first and second round of propagation.

When this analysis was sent to public health authorities in the form of a report, we hoped it could help health authorities and decision-makers regarding the best course of action and how to better allocate their resources. One application was the identification of indigenous populations in Brazil at imminent risk. In response, the indigenous communities organized themselves and in collaboration with other organizations, prepared a document for the UN. These subsequent reports are available in covid-19.procc.fiocruz.br.

In 9th July 2020, 110 days after the analysis here presented was finished, the paper was still under revision. During this period, SARS-CoV-2 spreaded throughout Brazil, causing 1.72 million confirmed cases and 68055 deaths. So, we extended this paper, giving an update of the situation, to compare our predictions with what happened.

After Rio de Janeiro and São Paulo, the next cities to reach three figures in their reported cases where Belo Horizonte/MG, Brasília/DF, Porto Alegre/RS, Salvador/BA and Fortaleza/CE. These cities have small effective distances to Sao Paulo, and for that reason, with high probability of outbreak according to our models (S1, S3, S4 Figs). Fast contamination of cities directly connected by road to Sao Paulo and Rio de Janeiro was also observed, mainly to the former. One month later, by the end of May, COVID-19 activity was already present in many cities along the coast as expected. These patterns had been predicted by the model.

We detected that micro-regions in the Northeast, in particular Ceará and Bahia, should receive attention due to the synergistic effect of geographical and social vulnerabilities on the magnitude of the COVID-19 public health crisis. Ceará was later strongly hit by the epidemic and had to implement lock down measures when hospital beds became unavailable.

Our model underestimed the spread of the epidemic within the Amazon region. This is due to the fact that human mobility by boat, the main mode of travel in the region, was not well represented in our mobility matrix. Thus, we did not predict the fast spread to communities along the Amazon river between Manaus and Belem.

Knowing in advance which regions could potentially suffer the biggest hit first might have allowed authorities to opt for preemptive differential investments to the public health care system (SUS) in these regions. Unfortunately, the initial efforts by the government to allocate resources in a rational way was not timely enough and was eventually interrupted by political and economic reasons.

## Supporting information

S1 File Additional information on the cluster analysis and complete results per micro-region. (DOCX)

S1 Fig Probability of outbreak for different values of R0 and the expected number of infected arrivals (*Im*). See text for details.
(PNG)

S2 Fig Map of COVID-19 cases in Brazil in 21-Apr-2020. (PNG)

S3 Fig Map of COVID-19 cases in Brazil in 21-May-2020. (PNG)

S4 Fig Map of COVID-19 cases in Brazil in 21-Jun-2020. (PNG)
