# Supplementary material for: Assessing the spread of COVID-19 in Brazil: Mobility, morbidity and social vulnerability
Source: PLoS One. 2020 Sep 18;15(9):e0238214. doi: 10.1371/journal.pone.0238214 (PMC7500629; doi:10.1371/journal.pone.0238214)
Supplement: S1 File — (DOCX) [file pone.0238214.s001.docx]

# **Supplement material: Predicting the potential impact of COVID-19 in Brazil: Mobility, Morbidity and the burden on the Health Care System**

# **High risk micro-regions in Brazil for COVID-19**

**S1 Table.** Micro-regions in Brazil with higher risk of immediate sustained transmission of COVID-19

| Micro-region | State | Effective distance to São Paulo | Social vulnerability | % elder population | Hospital beds per  10mil hab. |
| --- | --- | --- | --- | --- | --- |
| São Paulo | SP | 0.00 | A | 12 | 23.64 |
| Rio de Janeiro | RJ | 2.86 | A | 14 | 17.49 |
| Porto Alegre | RS | 3.54 | A | 13 | 26.99 |
| Brasília | DF | 3.59 | A | 8 | 60.21 |
| B Horizonte | MG | 3.68 | A | 11 | 22.78 |
| Salvador | BA | 3.86 | A | 9 | 26.81 |
| Curitiba | PR | 3.87 | A | 10 | 27.34 |
| Recife | PE | 4.03 | B | 11 | 32.76 |
| Florianópolis | SC | 4.25 | B | 11 | 30.55 |
| Fortaleza | CE | 4.33 | B | 9 | 27.03 |
| Goiânia | GO | 4.45 | A | 9 | 39.13 |
| Vitória | ES | 4.50 | A | 10 | 22.89 |
| Itajaí | SC | 4.58 | A | 10 | 16.12 |
| Cuiabá | MT | 4.73 | B | 8 | 28.46 |
| Maceió | AL | 5.13 | B | 9 | 25.77 |
| Foz do Iguaçu | PR | 5.13 | A | 10 | 25.06 |
| Campo Grande | MS | 5.17 | A | 10 | 25.91 |
| Uberlândia | MG | 5.25 | A | 11 | 18.57 |
| Manaus | AM | 5.28 | E | 6 | 16.85 |
| Natal | RN | 5.34 | A | 10 | 29.98 |
| Belém | PA | 5.41 | B | 8 | 21.69 |
| Porto Seguro | BA | 5.42 | B | 9 | 18.84 |
| Londrina | PR | 5.48 | A | 13 | 31.51 |
| Maringá | PR | 5.55 | A | 12 | 33.47 |
| Joinville | SC | 5.67 | A | 9 | 19.12 |
| João Pessoa | PB | 5.71 | B | 10 | 29.52 |
| Aracaju | SE | 5.71 | A | 8 | 26.55 |
| Ribeirão Preto | SP | 5.76 | A | 12 | 27.71 |
| S.José R.Preto | SP | 5.87 | A | 14 | 33.57 |
| Cariri | CE | 6.04 | C | 11 | 27.54 |
| Sudeste RR | RR | 6.08 | C | 6 | 16.40 |
| Teresina | PI | 6.09 | C | 9 | 32.93 |
| Pres.Prudente | SP | 6.20 | A | 14 | 30.15 |
| Chapecó | SC | 6.22 | B | 11 | 24.91 |
| Caxias do Sul | RS | 6.41 | A | 12 | 25.53 |
| Ilhéus-Itabuna | BA | 6.43 | C | 11 | 22.63 |
| Juiz de Fora | MG | 6.59 | B | 14 | 36.00 |
| Porto Nacional | TO | 6.63 | B | 6 | 35.72 |
| Porto Velho | RO | 6.66 | B | 6 | 33.32 |
| Petrolina | PE | 6.68 | C | 8 | 21.14 |
| Tubarão | SC | 6.73 | B | 13 | 24.31 |

**Table S2.** Micro-regions in Brazil with higher risk of immediate sustained transmission of COVID-19 *and* ***high social vulnerability***

| **Region** | **State** | **Micro-region** | **Social Vulnerability** |
| --- | --- | --- | --- |
| NORTH | AC | RIO BRANCO | C |
|  | AM | MANAUS | E |
|  | RR | BOA VISTA | E |
|  | RR | SUDESTE DE RORAIMA | C |
| NORTHEAST | PI | TERESINA | C |
|  | CE | LITORAL DE CAMOCIM E ACARAÚ | C |
|  | CE | SOBRAL | C |
|  | CE | ITAPIPOCA | E |
|  | CE | BAIXO CURU | C |
|  | CE | URUBURETAMA | C |
|  | CE | MÉDIO CURU | D |
|  | CE | CANINDÉ | D |
|  | CE | BATURITÉ | D |
|  | CE | CHOROZINHO | D |
|  | CE | CASCAVEL | C |
|  | CE | PACAJUS | C |
|  | CE | SERTÃO DE QUIXERAMOBIM | D |
|  | CE | LITORAL DE ARACATI | C |
|  | CE | BAIXO JAGUARIBE | C |
|  | CE | CARIRI | C |
|  | PB | CAMPINA GRANDE | D |
|  | PB | SAPÉ | D |
|  | PE | PETROLINA | C |
|  | PE | VALE DO IPOJUCA | D |
|  | PE | ALTO CAPIBARIBE | D |
|  | PE | MÉDIO CAPIBARIBE | D |
|  | PE | GARANHUNS | D |
|  | PE | BREJO PERNAMBUCANO | C |
|  | PE | MATA SETENTRIONAL PERNAMBUCANA | C |
|  | PE | VITÓRIA DE SANTO ANTÃO | C |
|  | PE | MATA MERIDIONAL PERNAMBUCANA | C |
|  | PE | ITAMARACÁ | C |
|  | BA | FEIRA DE SANTANA | C |
|  | BA | SERRINHA | C |
|  | BA | ALAGOINHAS | C |
|  | BA | ENTRE RIOS | C |
|  | BA | CATU | C |
|  | BA | SANTO ANTÔNIO DE JESUS | C |
|  | BA | JEQUIÉ | C |
|  | BA | VALENÇA | C |
|  | BA | ILHÉUS-ITABUNA | C |
| Southeast | MG | CONCEIÇÃO DO MATO DENTRO | C |
| South | PR | CERRO AZUL | C |
| Center-West | MT | ROSÁRIO OESTE | C |
|  | GO | CHAPADA DOS VEADEIROS | C |
|  | GO | VÃO DO PARANÃ | C |

#

# **Cluster analysis**

**Variables**

ESPVIDA : life expectancy at birth

MORTINF: Infant mortality

GINI: Gini index

PPOBRE: % of individuals below the poverty threshold

PEXPROB % of individuals below the extreme poverty threshold

RND1QTPOB: 20th percentile of the income

PCTAGUA: % individuals in households with access to piped water

PESGOINAD: % individuals in households with insufficient sewage disposal and water treatment

PSEMELITR: % individuals in households without electricity

PCTPOPURB: % of the population in urban areas

DHMEDU: DHM Education


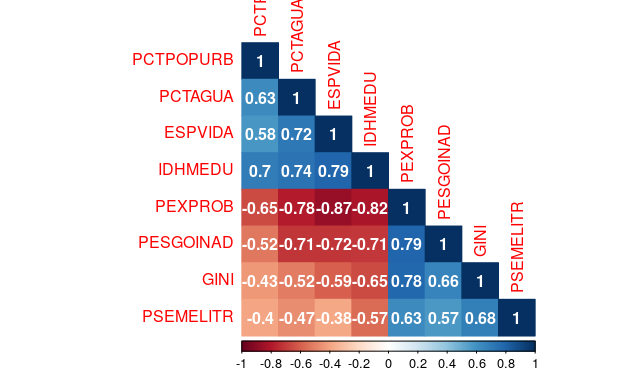


Figure S1. Correlation matrix of socio-economic indices that remained in the cluster analysis after removing the ones with high correlation. The only variable with high correlation that was kept is the percentage of individuals below the extreme poverty threshold. Despite the high correlation with life expectancy at birth and IDH edu, this variable was kept due to its importance as a descriptor of social vulnerability.


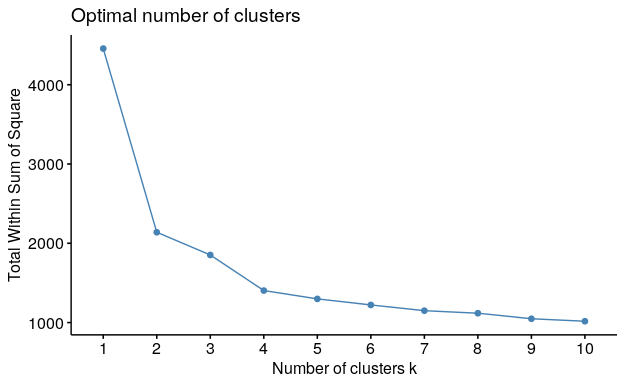


**Figure S2.** The best number of clusters according to the elbow method is four. However, after inspecting the spatial distribution of the micro-regions, k=5 was chosen to better discriminate poor regions in the Amazon and the Caatinga biomes.


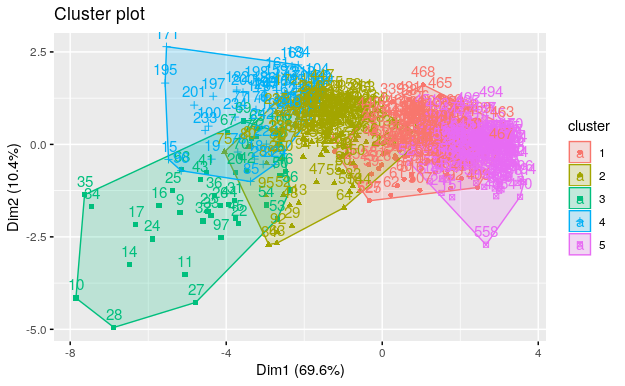


**Figure S3.** The first two components of the PCA explained ca. 80 % of the variance.
