# Supplementary figures and images for: Assessing the spread of COVID-19 in Brazil: Mobility, morbidity and social vulnerability
Source: PLoS One. 2020 Sep 18;15(9):e0238214. doi: 10.1371/journal.pone.0238214 (PMC7500629; doi:10.1371/journal.pone.0238214)

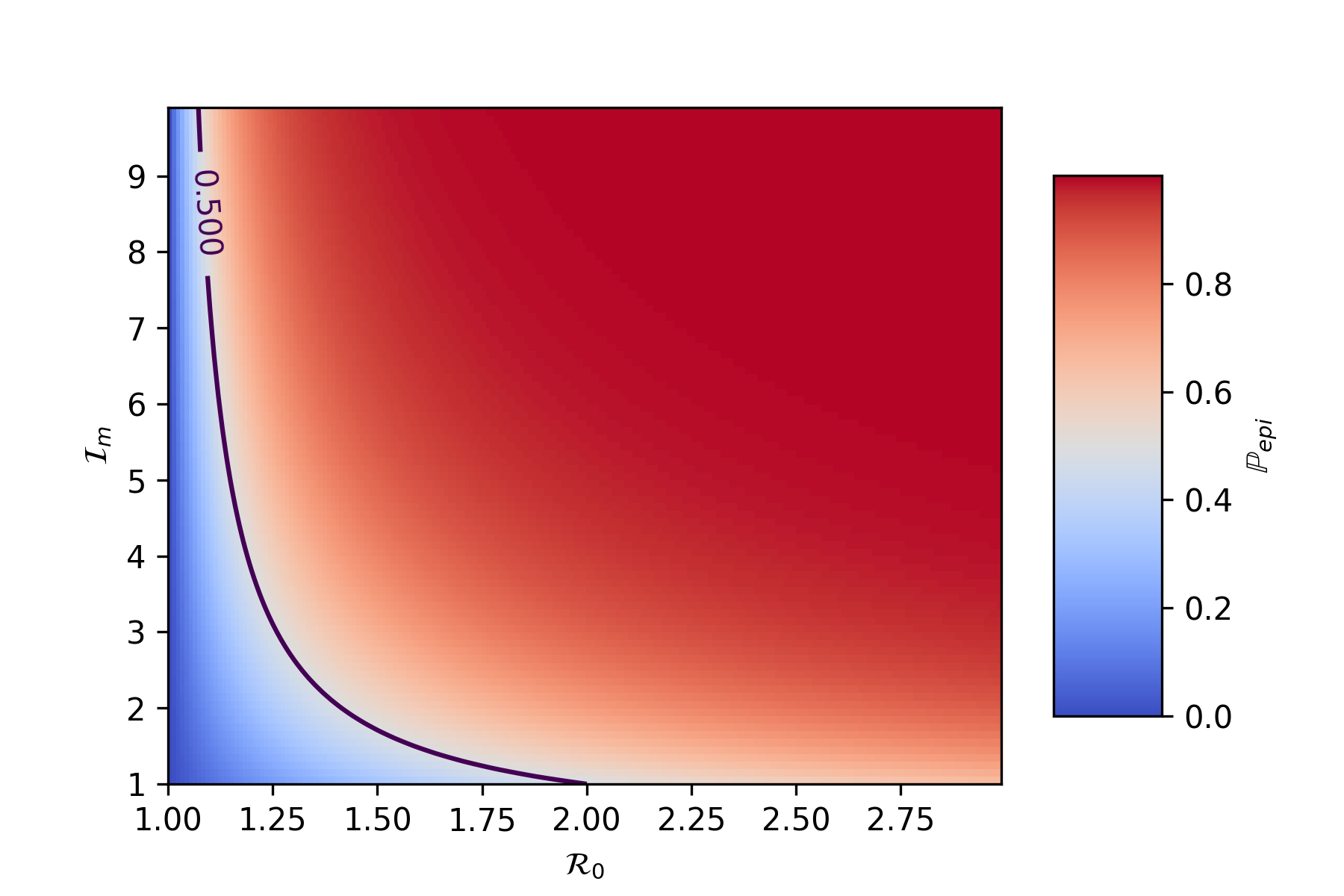

Supplement: S1 Fig — See text for details. (PNG) [file pone.0238214.s002.png]

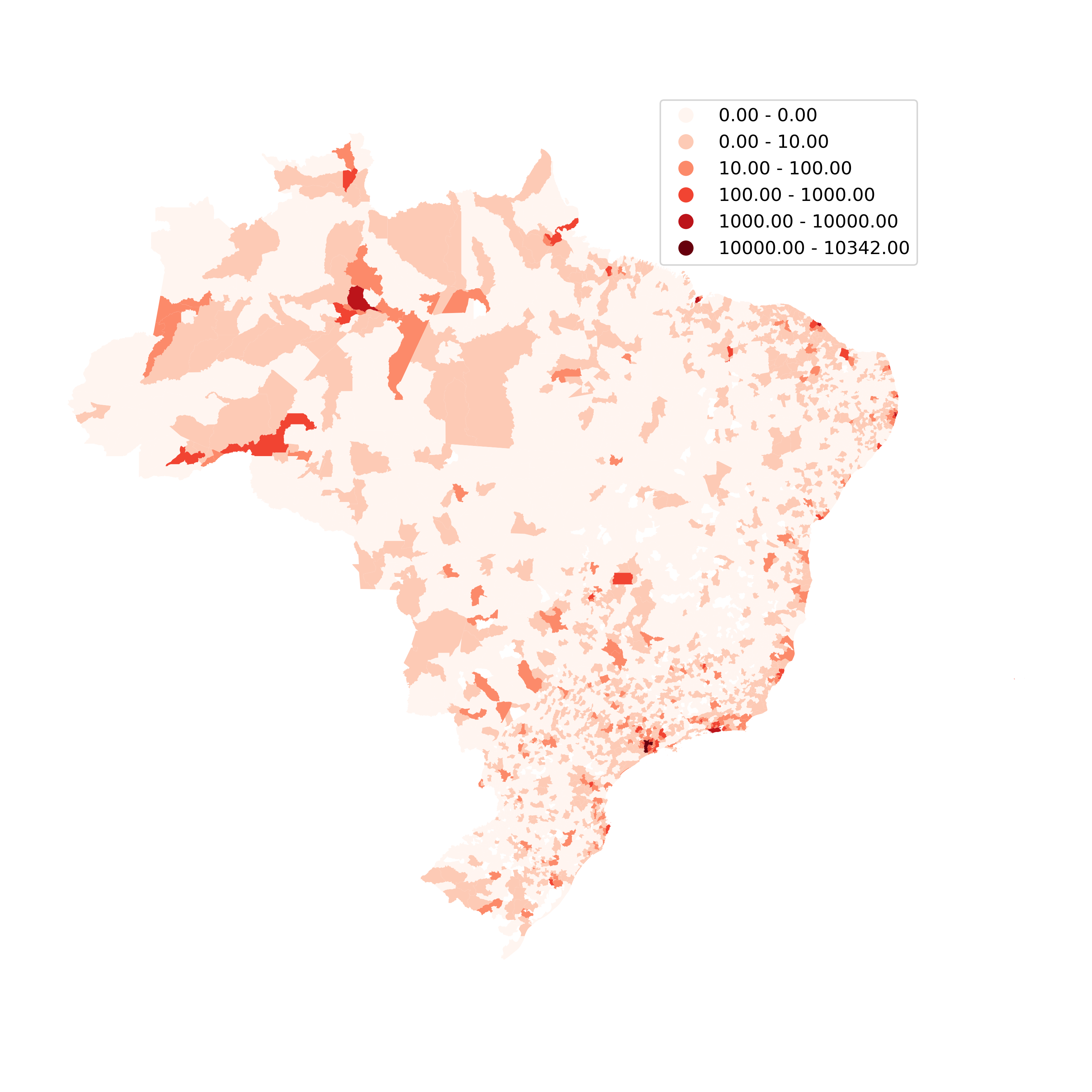

Supplement: S2 Fig — (PNG) [file pone.0238214.s003.png]

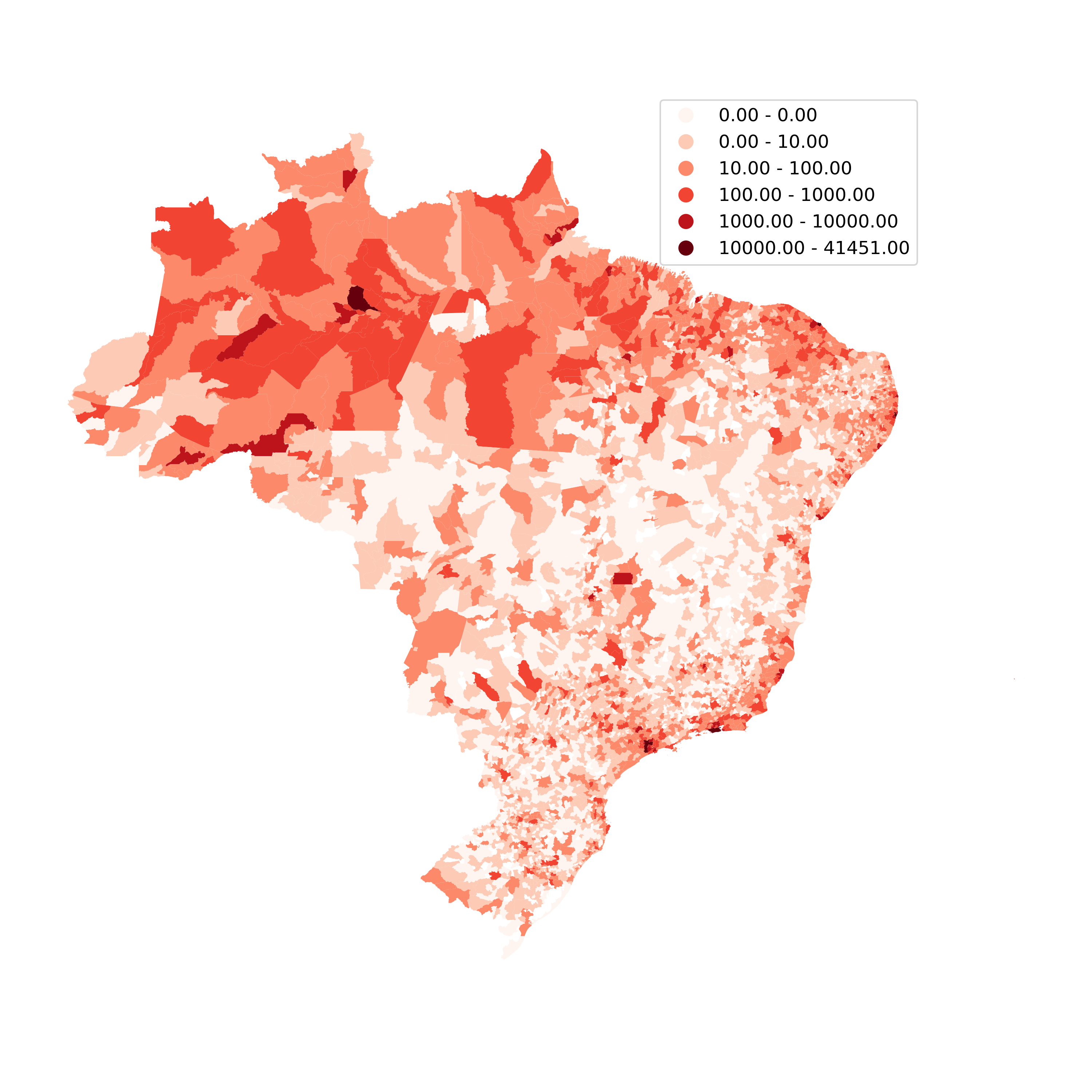

Supplement: S3 Fig — (PNG) [file pone.0238214.s004.png]

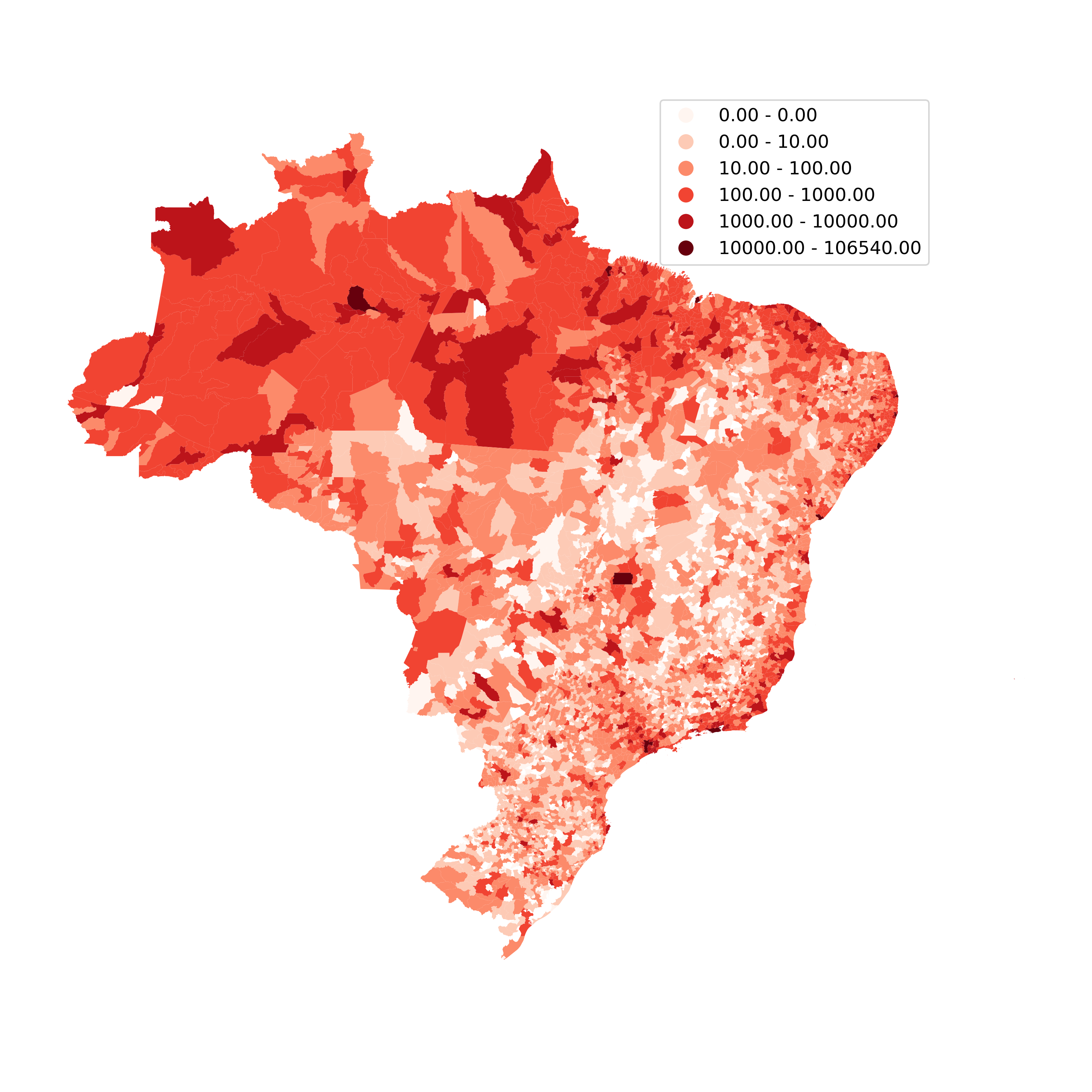

Supplement: S4 Fig — (PNG) [file pone.0238214.s005.png]
